# Evidence for the Effects of Xanthohumol in Disrupting Angiogenic, but not Stable Vessels

**Published:** 2007-12

**Authors:** Rita Negrão, João Incio, Rui Lopes, Isabel Azevedo, Raquel Soares

**Affiliations:** *Department of Biochemistry (U38-FCT), Faculty of Medicine, University of Porto, 4200-319 Porto, Portugal*

**Keywords:** angiogenesis, endothelium, matured vessels, neovessels, polyphenols, smooth muscle cells

## Abstract

Angiogenesis is a complex multistep process that comprises proliferation, migration, and anastomosis of endothelial cells, followed by stabilization of the newly formed vessel through the attachment of support cells. This process is imbalanced in a large number of disorders, including cardiovascular disease, diabetes and cancer. Evidence indicates that xanthohumol (XN), a prenylated chalcone present in beer, exerts anti-angiogenic properties. However, its precise effect within the angiogenic steps is not accurately established. The purpose of the present study was to examine which features of the angiogenic process can be disturbed by XN. Human umbilical vein endothelial cells (HUVEC) and human fetal aortic smooth muscle cells (SMC) were incubated with xanthohumol at 5 and 10 μM, and cell viability, apoptosis, invasion and capillary-like structures formation were examined. Treatment with 10 μM XN significantly decreased viability and invasion capacity and increased apoptosis in both cell types as assessed by MTT, double-chamber assay and TUNEL assay respectively. The two concentrations of XN further led to a significant reduction in the number of capillary-like structures, when HUVEC were cultured on growth factor reduced-Matrigel-coated plates. Interestingly, XN exhibited the opposite effect when HUVEC were co-cultured with SMC, leading to an increase in the number of cord structures. In addition, incubation of both types of cells with XN resulted in reduced activity of NFκB, a transcription factor implicated in these cell fates. Given the absence of adverse effects in mature vasculature by XN, these findings emphasize the potential use of XN against pathological situations where angiogenesis is stimulated.

## INTRODUCTION

Angiogenesis, the formation of new blood vessels, is a complex multistep process that involves extracellular matrix degradation, endothelial cell (EC) proliferation, migration and anastomosis, ending up by the recruitment and adhesion of pericytes or smooth muscle cells (SMC) that promote neovessel stability. This process is imbalanced in a large series of pathological situations, such as cardiovascular disease, psoriasis, diabetic retinopathy, rheumatoid arthritis and cancer (1). Evidence has been gathered regarding the effects of diet flavonoids in preventing angiogenesis (2-9). One of these molecules, xanthohumol (XN), is a prenylated chalcone present in beer (2, 4, 8-10). Epidemiological and experimental evidence indicates that XN is able to prevent proliferation and migration (2-4), rendering this agent a useful chemopreventive cancer agent. XN was found to exert anti-proliferative effects in human breast cancer MCF7 cells (10, 11) and in prostate epithelial cells (12, 13). Using human colon cancer cells, Pan *et al* (14) also showed that XN down-regulated bcl-2 expression, preventing, thus, caspase cascade activation. XN also exhibited anti-oxidant activity and anti-inflammatory properties (2-4, 10), preventing tumour progression. Oxidative stress and inflammation are two processes that cope with angiogenesis (1, 2). Therefore, we anticipate that in agreement with the effects of other polyphenols, XN should also be capable of exerting anti-angiogenic effects. However, only a few reports focused on the direct effects of this polyphenolic compound in vascular wall cells. Albini *et al* (8) have recently elucidated the anti-angiogenic effects of XN on EC. These authors found that EC’s ability to proliferate and invade was effectively inhibited by XN at 5μM. Furthermore, XN also prevented formation of vascular networks by EC in matrigel-coated plates (8).

In order to obtain mature stable vessels, newly formed vascular structures, which are only formed by a thin layer of EC, must be covered by support cells. Therefore, smooth muscle cells proliferation and migration are essential features for the assembly of normal vascularisation. Despite several papers reporting the inhibitory effects of natural polyphenols in smooth muscle cells, i.e. by preventing SMC growth and adhesion (15, 16), to our knowledge there are no studies regarding the effects of XN on this type of cells. The vascular effects of polyphenols are relevant for preventing angiogenic vessels formation, a pertinent issue for their use as anti-angiogenic agents in the treatment of a huge number of disorders (2-7). Nonetheless, it is also important that these compounds do not affect stabilized blood vessels. The aim of the current study was to identify the effects of XN within the whole angiogenic process. Accordingly, the present study addressed cell viability, apoptosis, invasion and capillary-like structures formation in endothelium and vascular smooth muscle cell cultures and in co-cultures of both cell types.

## MATERIALS AND METHODS

Human umbilical vein endothelial cells (HUVEC) were obtained from ScienceCell Research Labs (San Diego, USA). Cells were used between passages 3 and 8 in this study. HUVEC were cultured in M199 medium (Sigma-Aldrich, Portugal) supplemented with 20% fetal bovine serum (FBS) (Invitrogen Life Technologies, Scotland, UK), 1% penicillin/streptomycin (Invitrogen Life Technologies, Scotland, UK), 0.01% heparin (Sigma-Aldrich, Portugal) and 30 μg/mL endothelial cell growth supplement (ECGS) (Sigma-Aldrich, Portugal), and maintained at 37°C in a humidified 5% CO_2_ atmosphere. Cells were seeded on plates coated with 0.2% gelatin (Sigma) and allowed to grow. Human fetal aortic smooth muscle (FLTR) cells (SMC) were kindly provided by Dr James Mc Dougall (Fred Hutchinson Cancer Research Center, Seattle, Washington, USA). FLTR cells are immortalized SMC, which retain much of the phenotype of normal adult aortic SMC. These cells exhibit no phenotypic changes after passage 30 (17, 18). Cells were used in passages 50 through 60. SMC were cultured in high glucose Dulbeco’s modified Eagle’s medium (DMEM). Cells were maintained in 10% FBS and 1% penicillin/streptomycin and cultured at 37°C in a humidified 5% CO_2_ atmosphere.

XN (Sigma Aldrich, Lisbon, Portugal) was dissolved in ethanol and then added to cell culture medium at a concentration of 5 μM or 10 μM, established according to its IC_50_ as previously described (19). XN was added to serum-free M199 medium containing endothelial cell growth supplement (ECGS) (HUVEC) and serum-free DMEM (SMC) during 24 h. Control cells were incubated with vehicle (ethanol). Ethanol concentrations were kept below 0.1% in every culture.

HUVEC and SMC were allowed to grow until 70-90% confluence and then incubated with XN or ethanol for 24 h. After the incubation period, cells were washed twice with PBS and subjected to MTT [3-(4,5-dimethylthiazol-2-yl)-2,5-diphenyl tetrazolium bromide] assay as previously described (11).

Cells (1 × 10^4^) plated in glass coverslips were grown for 24 h and then incubated with XN or ethanol for 24 h. TUNEL assay (Terminal deoxynucleotidyl transferase-mediated deoxyuridine triphosphate nick-end labeling) was performed using the *In Situ* Cell Death Detection kit (Roche Diagnostics, Basel, Switzerland), according to the manufacturing instructions (11, 20, 21).

The invasive cell behavior in the presence of XN was quantified *in vitro* using a double-chamber assay by counting the number of cells that invaded a Transwell BD-Matrigel basement membrane matrix inserts (BD-Biosciences, Belgium), according to manufacturer’s instructions. FBS was used as chemoattractant. Results represent the ratio between invading cells in XN treated cultures compared to invasion in control cultures for the same initial amount of cells cultured.

Matrigel assay was performed on growth factor reduced-Matrigel (GFR-Matrigel) (BD Biosciences, Belgium)-coated plates for 24 h as previously described (22). Briefly, Cells were cultured on GFR-Matrigel coated plates for 24 hours, in medium containing XN or vehicle (ethanol). When cultured on matrigel, endothelial cells assemble into capillary-like structures. The number of cord-like structures was then measured in an inverted microscope. Each cord portion between the ramifications was considered one cord unit. Mean values were obtained by evaluating the whole cultures of each well under the same treatment. Capillary-like structures formation was also evaluated in co-cultures of HUVEC with SMC. SMC were added 6 h after the establishment of cord-like structures by HUVEC. Treatments were performed as described above. A semi-quantitative measurement of cord formation in GFR-Matrigel cultured HUVEC and HUVEC and SMC co-cultures was developed (tube formation index) as previously described (22).

NFκB activity was determined by ELISA assay. Nuclear extracts were prepared from HUVEC and SMC cells using the Nuclear extraction kit (Active Motif, USA). NFκB activity was measured using TransAM NFκB p65/p50 transcription factor assay kit (Active Motif, CA, USA). In brief, nuclear extract samples (5 μg) were added to a 96-well plate with immobilized oligonucleotide containing the NFκB consensus site. Sample wells were incubated with NFκB p65 subunit primary antibody, followed by incubation with HRP-conjugated secondary antibody. Quantification was performed at 450 nm and 650 nm using a plate reader (Thermo Electron Corporation, Multiskan Ascent, USA).

All experiments were performed in triplicate. Quantifications are expressed as mean (± SEM) of 3 independent experiments and are expressed as percentage of control, which was considered to be 100%. Statistical significance of difference between various groups was evaluated by one-way analysis of variance (ANOVA test) followed by the Bonferroni test. For comparison between two groups, Student’s t-test was used. A difference between experimental groups was considered significant with a confidence interval of 95%, whenever *p*<0.05.

## RESULTS

### Effects of XN in HUVEC

**Figure 1:**
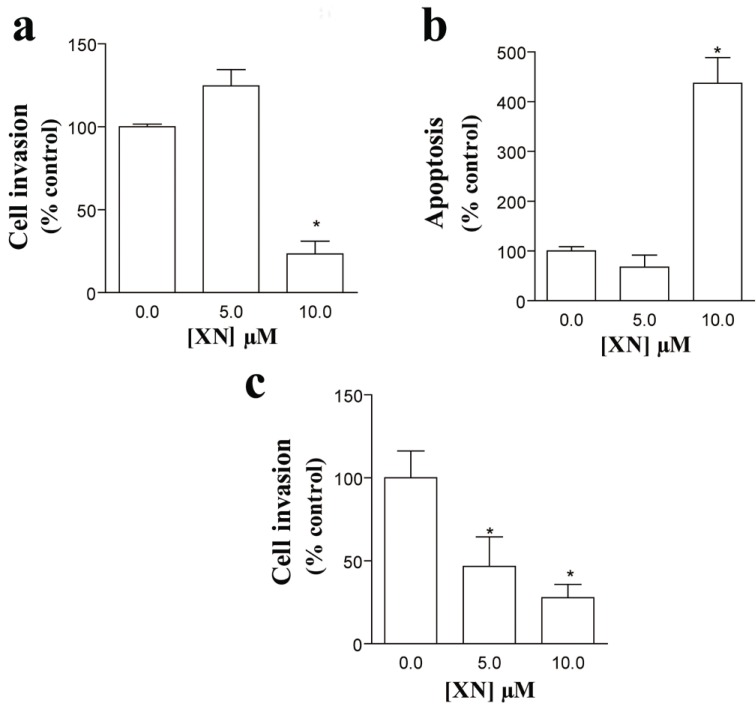
Effects of xanthohumol (XN) in HUVEC viability, apoptosis and invasive capacity. **(a)** Reduced cell viability after incubation with 10 μM XN, as evaluated by MTT assay (*p<0.05 vs. Control). XN at 5 μM did not significantly affect HUVEC viability. Results are expressed as percentage of control cells. Bars represent mean (SEM); **(b)** Increased percentage of apoptotic cells after incubation with 10 μM XN (*p<0.05 vs Control). No significant results were found upon 5 μM XN treatment relative to controls. Bars represent the percentage of apoptotic cells evaluated by the ratio between TUNEL-stained cells and DAPI-stained nuclei in every culture; **(c)** Effect of XN in HUVEC invasion. Incubation with XN resulted in decreased cell invasion in a dose-dependent manner (*p<0.05 vs. Control). Bars represent the percentage of invading cells relative to the initial amount of cells cultured.

To investigate whether XN exerted any effect on endothelial cells, HUVEC were incubated with 5 or 10 μM XN and cell viability was assessed by MTT assay. Although no change in HUVEC viability was found upon treatment with 5 μM XN, a significant reduction in viable cells was observed after incubating these cells with 10 μM (23.28% ± 7.77%, *p<0.05 vs. control) (Figure 1a). These findings were confirmed by a strong increase in the percentage of apoptotic cells after incubation with 10 μM XN (436.67% ± 52.03, *p<0.05 vs. control) (Figure 1b) as evaluated by TUNEL assay. Again, the number of apoptotic cells was not affected by 5 μM XN (Figure 1b). We next investigated whether XN affected migration and invasion capacity of HUVEC using a double-chamber assay. As illustrated in Figure 1c, XN resulted in a significant decrease in the migratory capacity of HUVEC in a dose-dependent manner (46.67 ± 17.79 and 27.85 ± 7.98 for 5 and 10 μM respectively; *p<0.05 vs. control).

### Effects of XN in SMC

**Figure 2:**
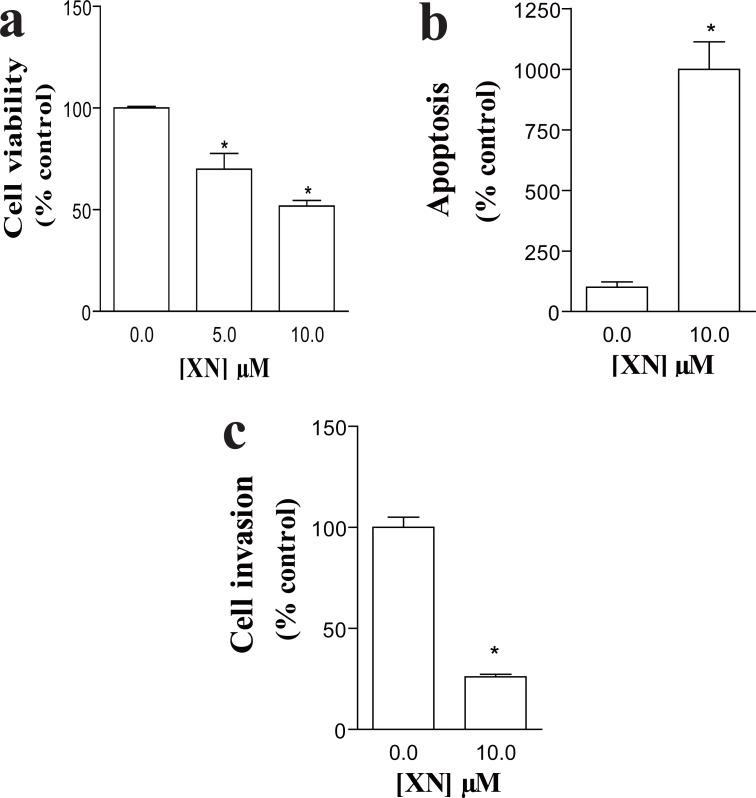
Effects of xanthohumol (XN) in SMC viability, apoptosis and invasive capacity. **(a)** Decreased cell viability after incubation with 5 μM and 10 μM XN as evaluated by MTT assay (*p<0.05 vs. Control). Results are expressed as percentage of control cells. Bars represent mean (SEM); **(b)** XN treatment significantly increased apoptosis as examined by TUNEL assay (*p<0.05 vs. Control). Bars represent the percentage of apoptotic cells evaluated by the ratio between TUNEL-stained and DAPI-stained nuclei in every culture; **(c)** XN resulted in effective reduction in invasive capacity as quantified in a double-chamber assay (*p<0.05 vs. Control). Bars represent the ratio between invading cells and the initial amount of cells cultured. Assays were repeated three times and performed in duplicate.

After the angiogenic process takes place, the endothelial cell layer of the new vessel stimulates the formation of a basement membrane and the attachment of support cells. This process is essential for the normal function of the neovessel, since angiogenic vessels, which are only formed by EC, often regress (1, 22). We, thus, evaluated the effects of XN in SMC viability, apoptosis and capacity to invade. A decrease in SMC viability was observed after incubation of VSMC with 5 (69.9% ± 0.76%, *p<0.05 vs. control) and 10 μM XN (51.8% ± 2.76%, *p<0.05 vs. control) as analyzed by MTT (Figure 2a). According to our findings and in agreement with published work (11, 13), the following studies were performed using XN at 10 μM concentration. Incubation with 10 μM XN resulted in a significant increase in the percentage of apoptotic cells by TUNEL assays (999.43% ± 113.99%, *p<0.05 vs. control) (Figure 2b). SMC were then cultured in inserts in serum-free medium and invasive capacity was evaluated in double-chamber assays. FBS was used as chemoattractant in the lower chamber in the presence of XN or ethanol. Treatment with XN resulted in a drastic reduction in SMC invasive ability as compared to controls (26.00% ± 1.30%, *p<0.05 vs. control) (Figure 2c).

### Effects of XN in cord-like structures formed by co-cultures of HUVEC and SMC

**Figure 3:**
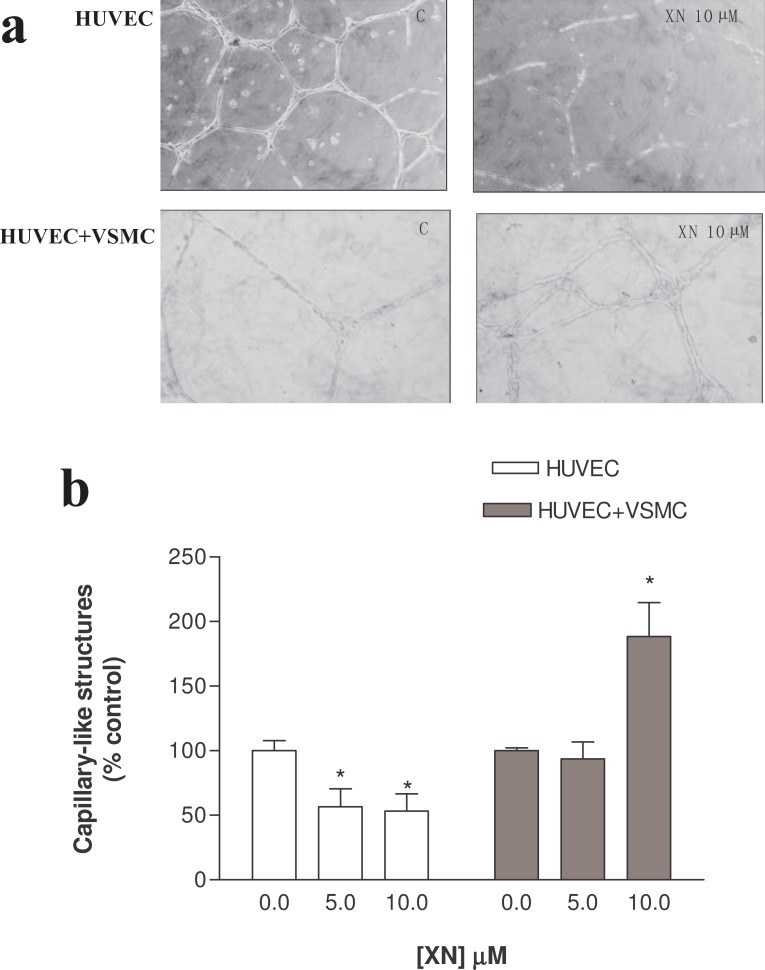
Capillary-like structures assembly was examined in HUVEC cultures or co-cultures of HUVEC and SMC after treatment with xanthohumol (XN). **(a)** In contrast to control cells (C), incubation with 10 μM XN resulted in unconnected structures presenting loosely edges, with many undifferentiated cells. The presence of SMC strengthens these cord structures (HUVEC+SMC). An increased number of these solid structures can be observed in the presence of 10 μM XN. Photos are representative of the whole cultures. Every culture was established in triplicate and visualized under an inverted microscope (x40 magnification); **(b)** Semiquantification of the tube formation index in HUVEC after incubation with XN at 5 or 10 μM (white bars). Reduced number of capillary-like structures formed upon incubation with 5 μM and 10 μM (*p< 0.05). Tubule-like structures formed by HUVEC and SMC co-cultures (dark bars) were enhanced in the presence of 10 μM XN (*p< 0.05 vs. Control). Bars correspond to the percentage of the number of tubule-like structures comparatively to controls. Error bars represent SEM between different assays.

To form a new blood vessel EC must assemble into vascular capillary structures. HUVEC are able to assemble into highly branched capillary-like structures when cultured on GFR-Matrigel. Therefore, we next examined whether XN was able to affect the formation of these structures. Incubation of HUVEC with ethanol (control, C) during 24 h, led to the formation of highly ramified cord-like structures (Figure 3a). However, the presence of XN at the two concentrations led to a drastic decrease in the number of these cord-like structures to 56.56% ± 13.95% (XN 5 μM) and 53.27% ± 13.25% (XN 10 μM) of control values (*p<0.05 vs ethanol) (Figure 3a and b). Ramifications were rarely found with loss of differentiated cells in the edges. These findings indicate that even at concentrations as low as 5 μM, XN was able to inhibit vessel assembly, a crucial feature for the angiogenic process.

This prompted us to investigate whether this agent also prevented the assembly of cord structures formed by co-culturing HUVEC together with SMC. We addressed this question by adding SMC after the assembly of HUVEC into capillary-like structures. In the absence of any treatment, HUVEC and SMC co-cultures displayed strings of branched tubule-like structures, which were formed by HUVEC surrounded by SMC. Incubation of these co-cultures with 5 μM XN for 24 h had no effect on the number of ramified structures as compared to ethanol-treated co-cultures (Figure 3b). Remarkably, the number of capillary-like structures was strongly increased by incubating co-cultures with 10 μM XN (188.40% ± 26.37%, *p<0.05 vs. ethanol) (Figure 3a and b) relative to controls, indicating that XN at this concentration enhanced the assembly of stable vessels.

### XN inhibited NFκB activity in both types of cells

**Figure 4:**
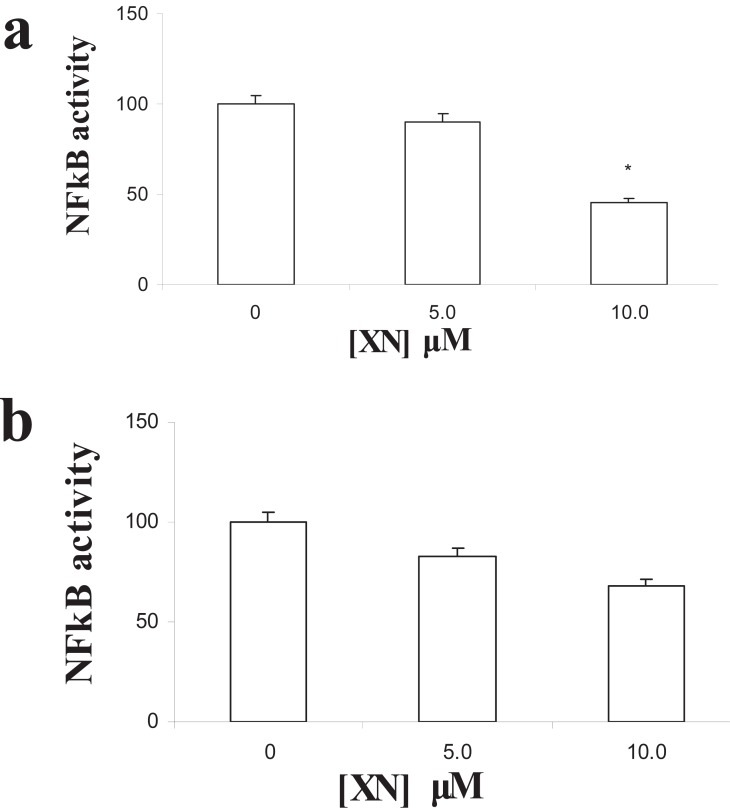
Effects of XN in NFκB p65 subunit activation in HUVEC and SMC. **(a)** A significant reduction in NFκB activity was found in HUVEC treated with XN in comparison to controls (ethanol) (*p< 0.05 vs control); **(b)** A tendency towards significant down-regulation of NFκB activity was found in SMC upon treatment with XN as compared to controls. Equal amounts of protein were loaded. Results are mean (SEM) of three independent experiments performed in triplicate.

NFκB is a transcription factor involved in many cell fates, including cell growth, apoptosis, migration and stimulation of inflammatory factors (1, 23). The broad effects of XN in vascular wall cells promted us to examine whether the activity of this factor was affected by XN in HUVEC and SMC by ELISA. A decrease in NFκB p65 subunit activity was found in HUVEC after incubation with XN in a dose-dependent manner, reaching statistical significance after treatment with 10 μM XN concentration (Figure 4a) (*p<0.05 vs. ethanol). A tendency towards an association was also found in SMC after incubation with the same concentration of XN (Figure 4b) (*p*=0.06 for 10 μM XN vs. ethanol), indicating that NFκB signalling inactivation is one of the pathways triggered by XN in these two vascular wall cells.

## DISCUSSION

Angiogenesis involve several modifications both in EC and in SMC (1). Natural polyphenols, including XN, are known to exhibit anti-angiogenic properties (5-9). However, the precise effects on the angiogenic process that are targeted by XN have not been clearly established. Herein, we examined the effects of this polyphenolic compound, in the whole angiogenic course, namely investigating cell viability, migration, invasion and capillary-like structure formation using both EC and SMC.

Incubation with XN at 10 μM resulted in a drastic reduction in the percentage of viable EC as compared to ethanol-treated cells. These results were expected, since anti-proliferative and pro-apoptotic effects of XN have long been described in cancer cells (3, 4, 11), implying that a similar mechanism is probably occurring in EC as well. Corroborating our findings, EC growth and apoptosis were previously reported to be disturbed by XN in tumour angiogenesis (8). These authors pointed out that incubation with XN at 5-15 μM resulted in a slight increase in apoptosis, whereas a high apoptotic rate was found in the presence of 25 μM XN (8). In the present study, a 10 μM concentration of XN was enough to induce a 4-fold increase in apoptotic rate as compared to ethanol-treated HUVEC. The disparity among the two studies is likely due to the fact that serum-free conditions were used in our experiment, which led to a more significant outcome.

Identical findings in cell viability were obtained in SMC under the same concentrations of XN. SMC constitute the media layer of the blood vessels wall. These cells attach to the basal membrane and promote vessel stabilization. SMC proliferation is a crucial feature for the establishment of mature blood vessels. The absence of SMC in blood vessel wall results in vascular leakage and frequent disruption (1, 24). Furthermore, SMC proliferation is associated with several disorders, including atheroma plaque formation and restenosis (1, 24, 25). XN was able to significantly reduce SMC viability and increase apoptosis in the current study. To our knowledge, this was the first report concerning the effects of XN in SMC. Given the established association between SMC viability and migration and disorders such as restenosis and atherosclerosis, our findings suggest that this polyphenol might be a putative therapeutic agent.

EC invasiveness is another mainstay in angiogenesis. Invasive capacity requires extracellular matrix degradation and involves the activation of EC invasive signalling pathways. Therefore, our finding that XN reduced EC invasion in a dose-dependent manner, indicates the relevance of this compound as an anti-angiogenic agent. Accordingly, XN has been reported to inhibit matrix metalloproteinase-2 release by HUVEC at these concentrations (8). Nevertheless, this reduction in invasive capacity was not restricted to HUVEC. Rather, an effective decrease in matrigel invasion was found in XN-treated SMC in the current study, demonstrating that this compound exerts a huge amount of effects on distinct cells, including vascular wall cells.

Ultimately, EC must assemble into capillary-like structures, in order to form a new blood vessel. We were able to show that XN prevented the formation of these structures on matrigel-coated plates in the two concentrations examined as compared to controls, implying that EC differentiation into cord structures is also affected by this natural polyphenol. Most strikingly, XN was not able to abrogate the assembly of capillary-like structures when HUVEC were co-cultured with SMC. In contrast to the effect found in HUVEC cultures alone, the number of capillary-like structures was not significantly changed by treatment with 5 μM XN in comparison to controls. Interestingly, the number of cord structures doubled in co-cultures incubated with 10 μM XN. Knowing that cord structures on matrigel assay are prone to disruption after 24 h culturing, the increase in cord structures found upon 10 μM XN incubation was attributed to the ability of XN in preventing vessel disruption. SMC bind to extracelular matrix proteins and to EC receptors, resulting in stabilization of blood vessels (1, 26). A series of receptor kinases including transforming growth factor-α, platelet–derived growth factors and angiopoietin-1 signalling pathways become activated by the adhesion of SMC to angiogenic vessels, resulting, thus, in vessel maturation (1, 26). According to our findings, 10 μM XN is likely to activate transduction pathways involved in SMC adhesion, enhancing, therefore, stabilization of blood vessels. This is a novel finding concerning the effects of XN in co-cultures of EC and SMC.

An increasing number of studies regarding the signaling pathways triggered by polyphenols in vascular wall cells have been reported (8, 15, 27, 28). Accordingly, immediate effects of apigenin and quercetin have been attributed to increased nitric oxide synthesis, which improved endothelial dysfunction (27, 28). Furthermore, XN inhibited endothelial NFκB activity, interfering with several intracellular phosphorylation cascades implicated in cell proliferation, migration and anastomosis (1, 12, 23). NFκB is an inflammatory promoter also involved in proliferation and down-regulation of apoptosis (1, 12, 23). The current paper shows that chronic treatment with XN was able to affect several processes within endothelial and smooth muscle cells. These findings led us to examine whether XN had any effect in NFκB activity in both types of cells. A reduced NFκB activity was found in both cells, reaching statistical significance in HUVEC. These findings are in agreement with other studies in the literature (12), and explain the large effects of this polyphenol in angiogenesis. In accordance, Imhof and Aurrand-Lions (29) hypothesized that NFκB was able to induce angiopoietin-2 (Ang2) in endothelial cells, an angiogenic factor that together with VEGF led to angiogenesis stimulation. Therefore, inhibition of NFκB signaling by XN probably results in abrogation of Ang2, explaining the underlying anti-angiogenic effects of this compound. In contrast, the presence of pericytes or SMC resulted in expression of angiopoietin-1, an Ang2 counterpart, preventing angiogenesis, even in the presence of NFκB (29). Accordingly, in co-cultures of HUVEC together with SMC, XN was unable to prevent capillary-like structure formation. Further studies focused on the inferred pathways induced by XN in these conditions are mandatory. Nonetheless, these findings clearly show that XN effects on vascular wall cells are restricted to angiogenic vessels, providing further evidence for the use of XN as a therapeutic strategy against dysfunctional vessels, whereas not distressing stable ones.

In conclusion, we were able to show that XN, a polyphenol present in diet, exerts a wide range of inhibitory effects in angiogenesis. Namely, XN prevents EC viability, invasion and capillary-like structures formation, while increasing apoptosis of these cells. Additionally, we also showed for the first time that identical effects were described in SMC as well. One probable mechanism for the XN effects in angiogenesis is the reduction of NFκB activity, a well-established angiogenic and inflammatory factor. These broadened effects of XN render this polyphenol a good candidate against disorders widely established in the western world that comprise excessive angiogenesis, such as cardiovascular disease and cancer. Furthermore, the fact that XN also targets SMC as revealed in this study provides new evidence for the use of this agent in pathological situation exhibiting SMC hyperplasia.
